# Retraction: Assessing the potential of exogenous caffeic acid application in boosting wheat (*Triticum aestivum* L.) crop productivity under salt stress

**DOI:** 10.1371/journal.pone.0343179

**Published:** 2026-02-18

**Authors:** The PLOS One Editors

The *PLOS One* Editors retract this article [1] because it was identified as one of a series of submissions for which we have concerns about compromised peer review and compliance with PLOS policy on Manipulation of the Publication Process.

HM did not agree with the retraction. GHA, MJ, ZM, MA, and RI either did not respond directly or could not be reached.
